# Alignment in post-approval changes (PAC) guidelines in emerging countries may increase timely access to vaccines: An illustrative assessment by manufacturers

**DOI:** 10.1016/j.jvacx.2020.100075

**Published:** 2020-08-25

**Authors:** Nora Dellepiane, Sonia Pagliusi, Prashant Akut, Sebastian Comellas, Norbert De Clercq, Shubhangi Ghadge, Thierry Gastineau, Mic McGoldrick, Ida Nurnaeni, Lorenz Scheppler, Regulatory Experts Working Group

**Affiliations:** a QRB Consultants Sàrl, 33, Chemin de la Petite Fontaine, 1270 Trélex, Switzerland; b DCVMN International, Route de Crassier 7A, 1262 Nyon, Switzerland; c Former Serum Institute of India Ltd, India; d Sinergium Biotech, Argentina; e GSK Vaccines, Belgium; f Serum Institute of India Ltd, India; g Sanofi Pasteur, France; h Merck Sharp & Dohme, Corp, United States; i PT Biofarma, Indonesia; j Janssen Vaccines, Switzerland

**Keywords:** Vaccine access, Variations guidelines, Post-approval changes, Regulatory science, Changes classification, Regulatory convergence

## Abstract

A comparison of the regulations and guidelines from 33 countries, across different regions, on the requirements and procedures for the management of chemical, manufacturing and control (CMC) changes for vaccines, also known as post- approval changes (PACs), reveals significant variability and lack of predictability of timelines for regulatory review and approval. These shortcomings imply that multiple data packages have to be prepared for submission to different authorities, generating a complex regulatory environment. Moreover, the timelines for approval by individual national regulatory authorities are variable, which results in manufacturers keeping various stocks of vaccines produced in accordance with the various approved specifications and procedures, in the different countries. This can seriously affect timely availability of vaccine in those countries. The World Health Organization (WHO) guidelines on procedures and data requirements for changes to approved vaccines provide a consensual framework for alignment, but are still underused. Reliance on both the review and approval by the regulatory authority in the country of manufacturing, or on the review performed by other national regulatory authorities, recognized by WHO as stringent, or on WHO prequalification dossier, offer alternative ways forward. These and other options to improve the management of post-approval changes during the product lifecycle of vaccines are discussed in this report, and aimed at improving guidelines alignment and regulatory convergence to advance immunization equity and coverage.

## Highlights

• Assessment of regulations and guidelines from 33 countries reveals complexity.
• Variability of regulatory guidance for review and approval of manufacturing changes.
• Reliance on the regulatory approval in the country of origin offers a solution.
• Improving guidelines alignment & regulatory convergence will benefit public health.

## 1 Introduction

Vaccines are complex biological products often formulated with multiple antigens, with or without adjuvants, provided in liquid or freeze-dried presentations. Production cycles are lengthy, ranging from several months to two or more years. Large volumes are required to reach millions of people in national immunization programs worldwide. While the required manufacturing capacity can occasionally be achieved at a single site, frequently vaccines’ production requires several sites in the same or different countries. To increase capacity, global manufacturing franchises are now a common business practice, adding to logistic and regulatory compliance complexity.

The commercial phase of product lifecycle starts with supply to markets once the registration procedure is completed and the marketing authorization (MA) is granted. Timelines for registration vary significantly between countries and are often difficult to predict. Registration in multiple countries represents a challenge [1], [2], [3]; however, the challenge is not limited to the first registration submissions in the country of origin (where the authority responsible for the regulatory oversight of the product is based and the final drug product is originally released) and procuring countries (countries importing the final drug product), but rather increases after the MAs have been granted.

After receiving the MA, vaccines are delivered to large populations, which bring further knowledge about their effectiveness and safety profile, initially assessed under clinical trials usually involving a few thousand subjects. New knowledge, acquired through the use of the vaccine in the field, must be managed in a structured and planned way to enable continual improvement and state of control, and to encourage innovation.

Post-Approval Change (PAC) is the term used to refer to specific changes or variations that a manufacturer makes to an already approved product under a MA or license. Variations and post approval changes are synonyms, however in this paper we refer to them throughout as Post-Approval Changes or PACs.

Furthermore, the lifecycle of vaccines is more complex compared to small-molecule drugs due to their inherent biological complexity and the longer time on the market, as for example, some vaccines, such as measles, yellow fever or polio vaccines, can be on the market for over 50 years [4], [5], [6].

**Fig. 1:**
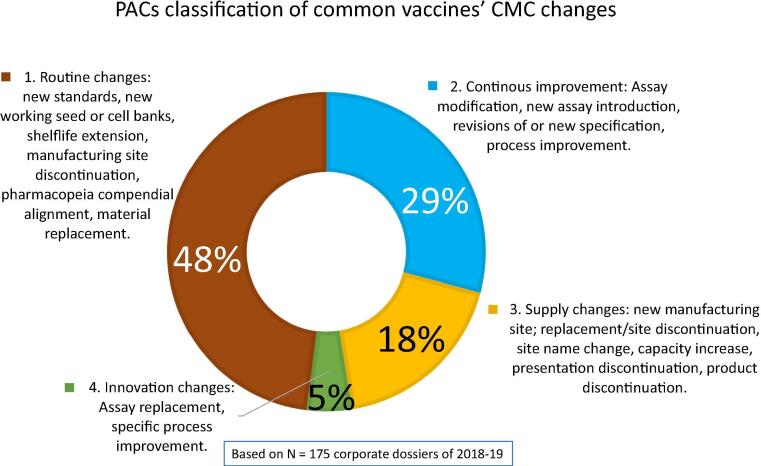
**PACs classification of common CMC changes.** The chart summarizes the proportion of most common types of chemistry, manufacturing and controls (CMC) introduced changes to vaccine products at post-approval stages. It illustrates four groups: 1. Brown: Routine changes include new standards, new working seed or cell banks, shelf life extension, manufacturing site discontinuation, compendial alignment, material replacement. 2. Blue: Continuous Improvement changes include assay modifications, new assays introduction, process improvement and revisions of specifications. 3) Yellow: Supply changes include introduction of a new site, site name change/discontinuation, capacity increase, presentation discontinuation, product discontinuation. 4) Green: Innovation changes include assay replacement, specific process improvements. Feedback from the field can drive changes like the need to discontinue a presentation, increase capacity, or specific changes linked to usage of the product. The data focuses on CMC changes only; it doesn’t contain clinical effectiveness and safety updates. The chart shows in average the percentage of changes that fall under each of these groups or categories based on 175 dossiers reviewed according to a large multinational vaccine manufacturer’s international experience, as to CMC changes only. The figure is a courtesy of GSK vaccines. (For interpretation of the references to colour in this figure legend, the reader is referred to the web version of this article.)

The main reasons for introducing changes to manufacturing are to enhance the robustness and efficiency of the manufacturing process or to improve the quality control methods used (as part of continuous process improvements), to respond to changes in regulatory requirements, or changes in suppliers or contractors utilized by the company, or to expand the manufacturing network for increased capacity to accommodate supply [7]. During a vaccine life cycle, PACs impacting the information covered by the marketing authorization dossier may need a submission to regulatory authorities worldwide. Regulatory guidelines require significantly more documentation for complex biological products than for drugs. Hence, chemistry, manufacturing and control (CMC) post-approval changes (PACs) submissions in many cases, require regulatory approvals that can be lengthy, delaying access in some countries to products manufactured through new or improved methods [8]. Fig. 1 illustrates the main reasons that typically trigger changes after initial approval of vaccines in each of four categories as a percentage of total corporate PACs dossiers assessed in 2018–19.

Thousands of PACs are submitted to regulatory authorities worldwide yearly for many different vaccines from many manufacturers. Some of these vaccines share the same antigen, in which case a single change to one antigen present in, for example, five different vaccines marketed in 150 countries could result in 750 filings. A single PAC approval can take around 2 years to get approvals in the majority of countries; however some outliers can take up to 4 years or more. In the meantime, a highly complex supply chain must be managed to ensure that the correct version of the vaccine is supplied to each country in accordance to the market-specific registration status.

Regulatory systems worldwide develop and evolve at different paces. In some countries, PACs are regulated while in other countries they are not. PAC regulations are divergent between different authorities with differences in classification, data requirements and implementation timelines. Some National Regulatory Authorities (NRAs) follow European [9], [10], USA [11], or other international guidelines, e.g. World Health Organization (WHO) guidelines [12], [13], [14], [15], while others have their own national guidelines for the review of PACs. Such divergence results in different implementation dates for changes, increasing the complexity in product logistics management until all PACs are approved in all countries. This complexity comes at a considerable cost and more importantly, negatively impacts the supply flexibility and security, hampering vaccination programs worldwide. Defined timelines for PACs’ approvals and compliance with them are a key element to secure sustainable vaccine supply as planned [8].

This study focused on the assessment of regulations and guidance documents from 33 different countries, regarding the classification of PACs and data requirements to support them, with the objective of raising awareness about the existing divergence and to make suggestions as to how PACs could be managed in a more aligned and predictable manner.

## 2 Methodology

The working group, comprised of regulatory and quality experts from the Developing Countries Vaccine Manufacturers Network (DCVMN) members and from the International Federation of Pharmaceutical Manufacturers and Associations (IFPMA), assessed publicly available regulations and guidelines regarding the requirements for review and approval of changes in a limited sample of 33 emerging countries from different regions, with available online information, which could be readily accessed and deemed sufficient to illustrate challenges, were selected (17 from Latin America, 8 from Africa and 8 from Asia) and three economic blocks. An online search was conducted to assess the available PACs guidelines following three patterns: guidelines from individual countries, guidelines from groups of countries (or economic blocks), and guidelines from individual countries based on adoption or reliance on WHO.

The assessment of countries’ guidelines focused on the following four types of guidance information:

A. Countries with own national guideline on PACs submission and management available
B. Countries with national guideline or PACs management procedure based on WHO Recommendations
C. Countries without specific PACs guideline, but information on PACs management is available in general registration regulation.
D. Timelines for PACs management officially stated in the PACs guideline or
E. Elsewhere (e.g. website).

The assessment of guidelines from 3 economic blocks, namely the European Union (EU), the Gulf Cooperation Countries (GCC) and the Eurasian Economic Union (EEU), focused on the classification approach, and timelines for review. It was not intended to do a comprehensive review of all available PACs guidelines worldwide.

## 3 Results

**Fig. 2:**
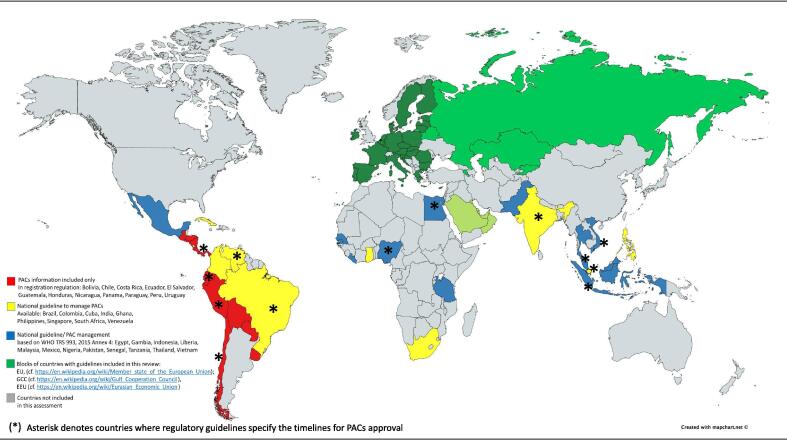
**Availability of PACs guidelines in a selected group of 33 emerging countries and 3 economic blocks.** The map shows the status of post-approval changes for selected 33 emerging countries and 3 economic blocks with respect to the availability of national guidelines on post-approval changes. It classifies them in 4 colour-coded groups: a) in yellow, emerging countries that have published own national guidelines which do not state to be based on any other international guideline including those recommended by WHO (Brazil, Colombia, Cuba, Ghana, India, Philippines, Singapore, South Africa, Venezuela); b) in blue, countries that rely or adopt WHO recommended guidelines or that have developed national guidelines based on those recommended by WHO (Egypt, Indonesia, Liberia, Malaysia, Mexico, Nigeria, Pakistan, Senegal, Tanzania, Thailand, The Gambia, Vietnam); c) in red, countries that do not have national guidelines but include guidance as to the management of PACs in their general registration regulations (Bolivia, Chile, Costa Rica, Ecuador, El Salvador, Guatemala, Honduras, Nicaragua, Panama, Paraguay, Peru, Uruguay); and d) in green, blocks of countries with guidelines, the difference in the tone of green indicates that the guidelines of different blocks are not necessarily similar (European Union = dark green; Eurasian Economic Union = green; Gulf Cooperation Council = light green). Singapore and The Gambia territory are so small that are not visible on a world map, therefore are represented as a circle. Countries in grey were not included in this assessment. The asterisk indicates countries that state in their guidelines, or in other published materials or documents, specific timelines to review and approve variations. The schematic representation therein does not imply the expression of any opinion whatsoever on the part of the authors concerning the legal status of any country, area or territory or of its authorities, or concerning the delimitation of its borders. The depiction and use of boundaries, geographic names and related data shown on maps and included in lists, tables, documents, and databases on this report do not imply any endorsement or acceptance. (For interpretation of the references to colour in this figure legend, the reader is referred to the web version of this article.)

The results of the assessment, based on the types of guidance available, are summarizes on Fig. 2. On the map, red corresponds to countries where a PACs guideline and information on PACs management is included in the medicines or vaccines registration regulations; in yellow, countries with national guidelines, but not necessarily based on WHO guidelines; in blue, countries with national guidelines which are based on WHO vaccine changes guideline [13]. Countries marked with an asterisk indicate where the timelines for PACs review and approval are officially stated/ published. This does not mean that these are in practice always followed. The figure shows also three economic blocks where guidelines on PACs are available, but these are not necessarily comparable or similar to each other.

The results of the assessment (Fig. 2) comprise 17 Latin American countries, four of which have developed their own national guidelines, namely Brazil [16], Colombia [17], Cuba [18] and Venezuela [19]. Other twelve Latin American countries have some guidance on how to manage changes in their respective registration regulations, i.e. Bolivia, Chile, Costa Rica, Ecuador, El Salvador, Guatemala, Honduras, Nicaragua, Panama, Peru, Paraguay and Uruguay [20], [21], [22], [23], [24], [25], [26], [27]. Only one country out of 17, Mexico, states in their guidelines that PACs regulation is aligned to guidance given in the WHO document [13]. The timelines for review of PACs are defined in 6 of the Latin American countries assessed, Brazil [16], Chile [21], Ecuador [22], Panama [23], Peru [24] and Venezuela [19]. For those with defined timelines, these vary between 30 days and six months depending on the classification of the change and the country. Although it was not part of the assessment, the Argentinean national medicines regulatory agency, ANMAT, has recently published a draft guideline for PACs on its website, though not officially implemented yet [28].

In Asia, six of the eight countries assessed here have national guidelines for PACs management. Pakistan regulatory agency has published on its website a draft PACs guideline [29], which seems not to be implemented yet. Except for the Philippines [30] and Singapore [31], all Asian countries assessed based their national guidelines on the WHO recommendations [13], including the draft guideline from Pakistan. India [32], Indonesia [33], Malaysia [34], Thailand [35] and Vietnam [36] have officially defined timelines for the approval of PACs. These vary between 40 days and six months depending on the classification of the change and the country. Vietnam assigns 15 days for notifications.

In Africa, from eight countries assessed, Egypt [37], Gambia [38], Ghana [39], Liberia [40], Nigeria [41], Senegal [42], South Africa [43] and Tanzania [44], have national guidelines for the management of PACs. Except for Ghana, all have their guidelines aligned to the WHO document [13]. Egypt and Nigeria have officially defined timelines for the management of PACs. Egypt guideline states 30 days for type I changes (minor) to give a first response, if additional information is required, it stops the clock for 30 days awaiting the response and issues a final decision within another 30 days. For type II changes (major) the guide states 20 days for the first response, allowance of 30 days to receive responses, if needed, and additional 60 days to issue a final decision [37]. Nigeria defines 30 days for immediate notification, 45 days for minor changes but does not give a timeline for major changes [41]. South Africa previously utilized the WHO guidance, but has recently adopted the EMA variation classification guidelines for human and veterinary medicines; they are planning to follow the EMA timing for variation approvals [43].

**Fig. 3:**
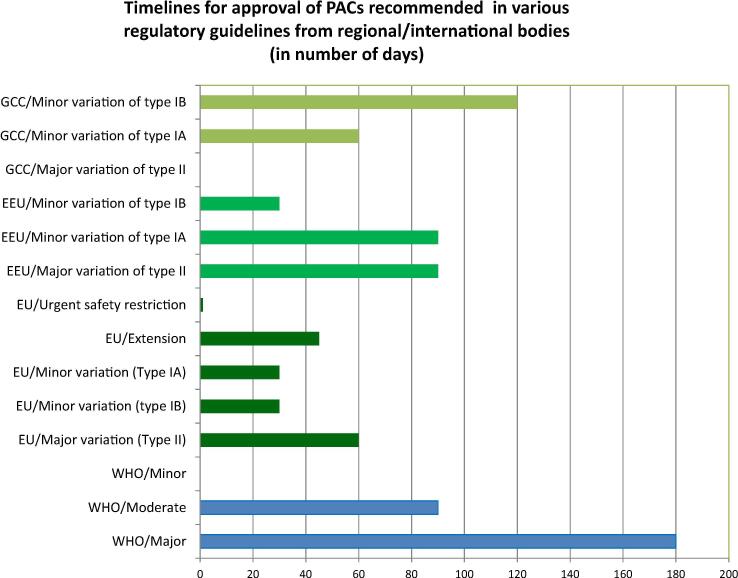
**Timelines for approval of PACs recommended in guidelines from 3 economic blocks and WHO.** The figure shows the classification of changes and the timelines taken for their review and approval for three regional economic blocks and for WHO based on the respective published guidelines. The Gulf Cooperation Council (GCC), classifies variations in minor of types IA or IB and in major or type II variations. It states timelines for review of minor variations only. The Eurasian Economic Union (EEU), classifies variations also in minor of types IA or IB and in major variations of type II, and provides timelines for review for all of them. In addition to the timelines defined for review of minor variations of type IA and IB and the major variations of type II, the European Union (EU), establishes timeframe for review of extensions to the marketing authorization and for urgent safety restriction. Variations of type IA have minimal or no impact at all on the quality, safety or efficacy of the medicinal product, variations of type IB are those that do not fall under a Type IA variation nor a Type II variation nor an Extension. Variations of type II are those that may have a significant impact on the quality, safety or efficacy of a medicinal product. In addition, an extension in EU and in EEU is defined as a variation which is listed in Annex I and fulfils the conditions laid down therein [10], [45]. Annex I lists three main categories, only two of which apply to human vaccines: 1. Changes to the active substance(s) 2. Changes to strength, pharmaceutical form and route of administration. An urgent safety restriction is defined as an urgent regulatory action triggered by the marketing authorisation holder or a national regulatory authority in the event of, or to prevent, a risk to human or animal health or to the environment. Both EU and EEU consider extensions and urgent safety restrictions in their variations guidelines, although only EU defines timelines for review.

Fig. 3 shows the timelines for approval of PACs recommended in guidelines from 3 regional economic blocks EU [9], [10], EEU [45], GCC [46] and for WHO [13]; the prescribed timelines for review depend on the classification of the change. Some blocks establish timelines for the review of major changes, such is the case of the EU (60 days for type II, which can be reduced to 30 in case of urgent needs and also extended to 90 days occasionally) and that of EEU (60–90 days for type II), whereas WHO [13] recommends 6 months for evaluation of a major change. The GCC guideline does not state timelines for major changes, while it does for moderate and minor changes, with 120 days established for a minor change type IB.

Five examples of post approval changes in vaccine manufacturing are described in Table 1, and how these changes are classified in different blocks and by WHO. Three of these changes, “Generation of a new Master Cell Bank (MCB)”, “Change in the manufacturing process of diluent” and “Qualification of a new reference standard” are not considered in national guidelines of one or more of the blocks chosen for this analysis. Changes in the manufacturing process of finished product, is considered as “major” in the regional blocks and as “moderate” in the WHO recommendations. Similar discrepancy is observed for a change in the manufacturing process of a diluent which is classified as a “moderate/minor” change by WHO, while it is not covered in the EU guidelines, and is considered as “minor” in the other two regional blocks. This variability in the classification leads to differences in requirements for approval of the change. Changes not covered in certain guidelines lead to the possibility of case by case management and therefore allows some divergence in requirements and unpredictable timelines for review.

**Table 1:** Comparative analysis of the classification of five selected examples of changes as per regional specific guidelines.

**Changes**	**Classification**			
	**WHO**	**EU**	**EEU**	**GCC**
Change in the manufacturing process of the finished product (excluding scale up)	Moderate	Major (Type II)	Major (type II)	Major (type II)
Generation of a new Master Cell Bank (MCB) from same /different expression construct	Moderate	Covered under B.I.a.2c Changes in the manufacturing process of the active substance Type II	Extension to Market Authorization	Not covered in the PACs guideline
Change in the manufacturing process of diluent	Moderate / minor	Not covered in the PACs guideline	Minor (type IB)	Minor (type IB)
Deletion of a diluent	Minor	Minor (Type IB)	Minor (type IB)	Minor (type IB)
Qualification of a new reference standard	Moderate	Not covered in the PACs guideline	Not covered in the PACs guideline.	Not covered in the PACs guideline

The table shows the classification assigned to five different changes in three economic blocks and by WHO, impacting the type of application (conditions and data requirements) and the respective timelines for evaluation.

## 4 Discussion

Only 11 of 16 of the regulatory guidelines from countries in Asia and Africa, assessed in our study, have based their national guidelines on the WHO guidance for vaccine post approval changes and variations [13], sometimes with minor adaptations. Still, this represents an important step towards convergence of regulatory standards and guidelines, which eventually could lead to increased alignment in requirements within regions and also in the future, to joint reviews and other regulatory mechanisms to further improve the efficiency of the product dossier evaluation processes. A good example of a guideline where the statements on timelines are transparently and thoroughly explained is the Egyptian guideline [37].

In Latin America, the situation is quite different: Four countries have developed their own national PACs guidelines. Because many countries do not have any regulations defining the timeframes, and only a few have timelines clearly stated in regulations, websites and/or other public information materials, timelines for review and approval of changes remain largely unclear and a challenge. Even for those countries where timelines are officially stated, these remain theoretical timeframes as per the regulations, and may not reflect the actual timelines, which can be much longer and often unpredictable.

For other authorities, statements about review timelines reflect their own handling time without accounting for “clock stops” and further time required to review responses from applicants. This lack of clock stop is one major reason why many countries do not comply with the stated timelines for review and approval of changes. Furthermore, in Panamá, Bolivia and Paraguay some major changes, such as transfer of a manufacturing facility, are considered as new products instead of a post approval change, and require a new submission for marketing authorization. IFPMA reported the timeframe taken by countries for the approval of a change to drug product specifications classified as “moderate”, which was one month in EU, four months in the USA and six months in Japan as prescribed in the regulation [9], [10], while in the rest of the world, nine percent of 53 other countries where the PAC was submitted, required national approval of the change, and for these countries the timeframe for approval varied between 3 and 38 months. Sixteen percent of the countries requiring national approval took more than 24 months to process the change submission [8]. Classification of changes, and timelines for regulatory review are major challenges in the management of PACs by regulators, and consequently in the management of product variations and supply logistics by manufacturers.

There is a need for consistency and predictability of PACs’ regulations and procedures to ensure that vaccines continue to be delivered timely, safely, reliably and efficiently to users around the world throughout their lifecycle.

The results of the assessment presented in this paper highlight the need for harmonization in guidelines based on an in-depth analysis of existing guidance and situation in 33 countries. The lack of clear guidance in many countries for certain PACs leads to uncertainties regarding their management. It also shows the lack of transparency regarding procedures in place and timelines for regulatory review in many countries and hence lack of predictability.

Although WHO has developed a set of guidelines on PACs management, the adoption of such guidelines at individual country level remains limited [12], [13], [14], [15]. Most countries have a regulatory system covering both vaccines and pharmaceuticals and single guidance documents covering all products. In addition, WHO has four guidance documents concerning variations: one for vaccines, one for biotherapeutics, one for pharmaceuticals and one for multisource pharmaceuticals [12], [13], [14], [15]. Implementation of a tiered, risk-based classification system for changes to MAs based on the principles outlined in the relevant WHO guidance [13], would represent a significant step forward towards global alignment.

The use of common classification systems, and clear and consistent timelines should be implemented for the regulatory assessment of post approval changes, specifically 3–6 months for major changes and 1–3 months for moderate changes, in line with WHO guidelines on post approval changes. Adherence by NRAs to the specified timelines for regulatory assessment is critical [8].

The ICH Q12 guideline, Technical and Regulatory Considerations for Pharmaceutical Product Lifecycle Management, is an example of an initiative that provides a framework to facilitate the lifecycle management of post approval of CMC changes in a more predictable and efficient manner [47]. The WHO guideline should be revised in the light of this new guideline as well as integrating risk-based approaches as described in the recent Parenteral Drug Association (PDA) publication on the Effective Management of PACs in the Pharmaceutical Quality System.^1^ Its revision might represent an opportunity to update the document on other points as well, such as the requirement for pre-approval of moderate changes, or changes where requirements are more restrictive than those in the EU guideline (e.g. changes of reference standards and those impacting facilities and equipment), triggering more stringent reporting in some countries compared to what is being done in countries of origin, such as in the EU.

Ideally, PACs should be reviewed by the regulatory authority in the country of origin and approved in all other countries based on this initial approval. Recognition and reliance on the work performed by the NRA responsible for the oversight of the product in country of origin should be the ultimate goal. The fact that there are different levels of maturity across NRAs worldwide is well known and contributes to insufficient use of reliance and recognition mechanisms among NRAs. The WHO prequalification (PQ) system recognizes this fact by applying different levels of reliance. Products manufactured in countries, where the responsible regulatory authorities are considered stringent (SRAs)^2^ are eligible for a streamlined prequalification procedure with heavy reliance by WHO on the reports issued by the relevant NRA. If, on the contrary, the NRA responsible for the product oversight is not considered to be a SRA, the product is subject to full and independent evaluation through the WHO PQ process and the reliance on the NRA is limited to specific post-marketing activities [48]. The concept of SRA will be gradually replaced by that of “WHO listed Authority” (WLA) [49].

Roth et al. in a study aimed at helping prioritize investments for a sustainable strengthening of medicines regulatory systems, identified three major regulatory challenges at global level [50]:

(a) implement value-added regulatory practices,
(b) timely access to new quality-assured medical products without compromising safety and efficacy
(c) limited evidence-based data to support post-marketing regulatory action.

One of their proposed strategies to address the point b) above, is to strengthen registration efficiency and timelines and one to address point c), is to strengthen regulatory management of variations. The authors note that in low- and middle-income countries (LMICs) often the resources and knowledge for variation management are not available which leads to delays in supply due to shortages of the approved product. Reliance on WHO PQ and/or mature NRAs is proposed as an efficient mechanism to ensure continued availability of quality products [50].

Although reliance on regulatory approval by the country of origin NRA, other WLAs or by WHO-PQ may be extremely important, providing together with the dossier the information of where the PAC has been approved, and the exchange of information that was held with the NRA may also be helpful, and facilitate the process.. Since the “mutual recognition” targets may still be some time away, some shorter term improvements may be considered.

In addition to developing more worldwide alignment of regulatory requirements and regulatory reliance mechanisms across regulatory agencies and with WHO, other elements would represent significant improvements for an efficient management of PACs. Some options that could be considered by regulatory authorities are proposed below:

• **Harmonization of administrative documentation**: country-specific administrative requirements add a significant amount of time for manufacturers to cope with each request. Harmonizing those requirements in such a way that the same set is provided to all countries could dramatically simplify the preparation of submissions and contribute to reduce the overall duration of the process for managing PACs;
• **Cross referencing**: a cross-referencing mechanism could be considered so that the product dossier is submitted only once with cross references to all impacted licenses; by doing so, only one review would be performed and the administrative burden reduced;
• **Grouping**: regulators should enable the possibility to bundle multiple changes under one and the same dossier, when they are connected, instead of requesting as many dossier submissions as PACs;
• **The WHO Collaborative Registration Procedure (CRP)**^3^ is a voluntary agreement between WHO, the NRA of country registering the product, and the manufacturer to replace the full review of the dossier by the review of the reports resulted from the prequalification evaluation by WHO, and limits the timeline for registration to 90 days. Extended use of the CRP would allow for a facilitated mechanism for approval of PACs in products that fall under the CRP umbrella;
• **Acceptance of Post Approval Change Management Protocol (PACMP)**: as already existing under the WHO guideline as well as triggered by the recently endorsed ICH Q12 guideline, the use of PACMP should be made possible across all countries provided they also introduce an appropriate lower reporting category to enable the reporting of results after executing the protocol. The current limitation in the use of this mechanism by the Industry, while representing a good regulatory process, is mainly linked to the fact that only a very limited number of countries accept this process.
• **Rolling submissions**: Being able to submit the dossier in parallel to the submission in country of origin and providing the approval from this country as it is granted would dramatically reduce timelines.

The regulatory approval of PACs is extremely complex. It has been recognized both by manufacturers and regulators as one of the contributing factors to supply issues and timely access of vaccines to populations that need them. One of the main causes of this complexity is the highly heterogenous international regulatory landscape, as many countries have their own requirements and processes.

Alignment of regulatory requirements on the basis of the WHO guideline on changes to approved vaccines [13], including classification, and timelines for review, would represent a significant improvement with respect to the current *status quo*. Moreover, compliance with timelines is also included in the assessment of Regulatory Authorities as part of the WLA process, which could be reinforced.

Taking action on these challenges, as a matter of urgency, is critical to address supply constraints linked to the regulatory challenges described in this study and would help to reach the populations in need with the required vaccines in a timely manner.

Recognition and reliance on the country of origin NRA represents the ideal mechanism to maximise the process efficiency, though not a short-term solution. The alternative solutions, which have been briefly discussed above, when used in combination, could lead to a more efficient use of resources, increased expertise of NRAs, as well as increased trust between authorities which could in turn facilitate establishment of collaborative and reliance mechanisms. Such alternative solutions would mitigate the current challenges and pave the way towards recognition of the regulatory oversight effected by the country of origin NRA.

## 5 Conclusion

To secure the timely supply of vaccines to the populations globally, the efficient management of PACs asks for prompt action with respect to:

i. alignment/harmonization of requirements (possibly based on the WHO guideline),
ii. reliance on established reliable mechanisms such as extended use of the WHO CRP,
iii. official establishment of timelines for review and approval of changes and compliance with such commitment,
iv. transparent communication of the procedures in place, and
v. combinations of the above proposed options or others that may be proposed, to reduce the number of PACs to be reported to NRAs.

Manufacturers are committed to the process and urge regulators globally to converge towards harmonization, based on the WHO guideline. PACs guidelines need to be revised to integrate the recently approved ICH Q12 principles, as well as above outlined ideas which are expected to dramatically simplify the growing complexities in regulation to enable populations to equally benefit of the latest high-quality vaccines across the world.

Every effort should be made to overcome the difficulties as indicated by the PAHO/WHO Country Representative in Brazil, Socorro Gross, in the frame of the recently signed MOU with BRICS^4^ countries: *“We seek international convergence in the area of health regulation while respecting the particularities of each country, with all its political, economic, social, cultural and geographical characteristics, because without this convergence the health of citizens around the world is compromised. Convergence can bring more innovation and faster and better product control”.*

## Declaration of Competing Interest

The authors declare that they have no known competing financial interests or personal relationships that could have appeared to influence the work reported in this paper.

## Footnotes

1 https://journal.pda.org/content/early/2020/05/28/pdajpst.2020.011734

2 WHO defines a SRA as a regulatory authority which is a member or an observer of ICH, or is associated with an ICH member through a legally binding mutual recognition agreement Working document QAS/17.728/Rev.1 August 2017.

4 BRICS countries (Wikipedia definition): **BRIC** is a grouping acronym referring to the group of **countries** of Brazil, Russia, India and China, deemed to be developing **countries** at a similar stage of newly advanced economic development, on their way to becoming developed **countries**.

## Footnotes

3 More information at https://extranet.who.int/prequal/content/collaborative-procedure-accelerated-registration
